# The London Quarterly Journal of Dental Science

**Published:** 1857-07

**Authors:** 


*The London Quarterly Journal of Dental Science.*—It gives us pleasure to announce the advent of a new quarterly devoted to the advancement of the science and art of Dental Surgery, and under the most favorable aus pices. Judging from the ability displayed in the present No., issued April 1st, 1857, it is certainly destined to hold a high position in the list of scientific journals.

We have reason to believe that this new periodical is under the editorial management of Dr. James Robinson, of London, a gentleman long and favorably known by his many able and valuable contributions to the literature of the dental profession. If our surmises be correct with regard to this matter, no fears need be entertained as to the ultimate success of this new candidate for professional honors. Dr. Robinson knows the wants of the dental profession, and, judging from what we have seen from his pen, he is not only a scientific, but also eminently a practical man—qualifications essential to the successful management of a publication like this. Most cordially, therefore, do we tender to our transatlantic contemporary the right hand of fellowship, sincerely hoping that its future, as we have no doubt it will, may realise the promise given by the number now before us.

We hope our professional brethren on this side of the Atlantic, will show their appreciation of this new enterprise by extending to it a liberal support. It is published quarterly, at £1, or $5, per annum, and it will give us pleasure to order it for any of our friends who may desire to take it.

